# Impact of Soft Tissue Pathophysiology in the Development and Maintenance of Bisphosphonate-Related Osteonecrosis of the Jaw (BRONJ)

**DOI:** 10.3390/dj4040036

**Published:** 2016-10-25

**Authors:** Thomas Ziebart, Frank Halling, Paul Heymann, Andreas Neff, Sebastian Blatt, Junho Jung, Andreas Pabst, Leonardo Righesso, Christian Walter

**Affiliations:** 1 Department of Maxillofacial Surgery, University Hospital, Baldingerstraße, D-35043 Marburg, Germany; Dr.Halling@t-online.de (F.H.); heymann.paul@gmail.com (P.H.); neffa@med.uni-marburg.de (A.N.); 2 Department of Maxillofacial Surgery, University Medical Center Mainz, Augustusplatz 2, D-55131 Mainz, Germany; sebastian.blatt@gmx.de (S.B.); ssa204@gmail.com (J.J.); andipabst@me.com (A.P.); righesso@protonmail.com (L.R.); walter@mainz-mkg.de (C.W.); 3 Oral and Maxillofacial Surgery, Mediplus Clinic, Haifa-Allee 20, D-55128 Mainz, Germany

**Keywords:** gingiva, bisphosphonate, soft tissue, fibroblasts, keratinocytes, bisphosphonate associated osteonecrosis of the jaws

## Abstract

Since the first description of bisphosphonate-related osteonecrosis of the jaw (BRONJ), numerous research groups have focused on possible pathological mechanisms including the suppression of the bone turnover of the jaw, antiangiogenic effects and soft tissue toxicity. In our review we focused on summarizing the role of the soft tissues in the development and progression of BRONJ. The biological behavior of fibroblasts can be significantly influenced by bisphosphonates (BP) such as a concentration dependent reduction of cell viability. High concentrations of BP can induce apoptosis and necrosis of the cells. Comparable effects could be detected for keratinocytes. Compared to non-nitrogen containing bisphosphonates, nitrogen-containing BP have worse effects on cell biology by blocking the mevalonate pathway. Further, the cell architecture and expression levels of several genes and proteins are significantly disturbed by BP. These inhibitory effects of BP are in accordance with BP-related reduced angiogenesis and neovascularization and could underline the hypothesis that inhibition of fibroblasts and keratinocytes results in delayed wound healing and can induce and trigger BRONJ.

## 1. Background of BRONJ

Bisphosphonates (BP) are widely used in different benign and malignant diseases such as Paget’s disease, osteoporosis, multiple myeloma and bone metastases of breast or prostate cancer. From 2003, Marx et al. and other international research groups have reported on a medication-associated osteonecrosis of the jaw and called it bisphosphonate-related osteonecrosis of the jaw (BRONJ) [1]. Previous studies report that the incidence of BRONJ ranges from 0.94% to 18.6% worldwide [2,3].

In recent decades, various international research groups have attempted to analyze the pathophysiology of BRONJ [4,5,6,7]. Besides the mechanism of the hard tissue disturbance, immune system disorders and anti-angiogenic effects, research has mainly focused on the effect of the soft tissue [5,8,9].

Apart from the inhibitory effect of bisphosphonates (BPs) on osteoclasts and osteoblasts, nitrogen-containing BP in particular interacts with cell soft tissue cells such as fibroblasts and keratinocytes [10,11]. After local accumulation of BPs, and especially in combination with other cancer medications such as chemotherapeutics und angiogenesis blocker, BPs might directly affect the oral mucosa via tissue toxicity. This could lead to gingiva injury followed by bone exposition, the main clinical sign of BRONJ [12,13]. Disturbed biological activity of the soft tissue could also result in delayed mucosal healing after tooth extraction or dentoalveolar surgeries in patients treated with BPs [14]. Therefore soft tissue management plays an important role in oral surgery intervention: After resection of osteonecrosis, a watertight coverage by good vascularized local tissue is mandatory [15]. In advanced stages of disease, the jaw can be rebuilt by microvascular flaps, e.g., the osseocutaneous fibular flap [16].

## 2. Characteristics of the Oral Mucosa

In comparison to other parts of the human body, the oral gingiva is unique showing special features. Unlike other epitheliums, the oral gingiva is in direct contact with the underlying bone. Under BP treatment, there is a direct cytotoxic effect by blood support as well as the BPs enriched underlying bone. No soft tissue layer, such as fat, fascia, or muscle, buffers the negative effect of BPs released from the underlying bone [17].

Under normal conditions, the mucosal immune system suppresses pathogenic organisms such as bacteria and fungi.

These physiological and anatomical factors are unique to the oral environment and may represent an important factor for the course of the BRONJ disease. Furthermore this explains the fact that oral mucosa is strongly inhibited by BPs. Different research groups additionally showed, in vivo and in vitro, that BP can directly counteract and inhibit cells of the immune system such as neutrophils and lymphocytes [18,19]. The missing link is seen in measurements of BP-concentration in oral soft tissue, which would support this theory.

## 3. Impact of Bisphosphonate on Fibroblasts

Collaboration between osteoblasts and osteoclasts is strongly required for normal bone turnover. This physiological link is disturbed in patients treated with BP [20]. Under normal conditions, a group of molecules including RANK-L, osteoprotegerin (OPG) and interleukin 6 (IL-6) are produced by osteoblasts [21,22]. BP-treatment disturbs the RANK-L-OPG-system and IL-6 expression in osteoblasts by decreasing the production of RANK-L and IL-6 [23]. In addition to cells of the immune system, e.g., T-cells, fibroblasts can produce RANK-L and OPG, too. Bacterial infection leads to inflammatory conditions by lipopolysaccharide (LPS). LPS has a direct effect on fibroblasts, which increase the production of Il-6 and RANK-L [24]. Fibroblast growth factor (FGF) is another important cytokine for bone metabolism. FGF induces BMP and RANK-L expression from osteoblasts [25]. Taken together, BPs counteract not only with osteoblasts and osteoclasts, they also influence bone turnover via inhibition of fibroblasts.

## 4. Impact of Bisphosphonates on Keratinocytes

For sufficient oral wound healing, viability of keratinocytes is mandatory. Reduction in cell viability may result in exposed bone, which could serve as the ignition spark for BRONJ. Pabst et al. demonstrated that nitrogen-containing BPs have a strong influence on keratinocytes. BPs decrease cell viability, migration ability, and increase apoptosis rate [26], i.e., keratinocytes interact with osteoblasts and osteoclasts by different cytokines. Via production of the epithelial growth factor (EGF), keratinocytes induce differentiation of osteoclasts and RANK-L expression by osteoblasts [27,28].

Taken together these studies support, but cannot categorically confirm, the theory that pausing the drug administration during oral surgical procedures could be beneficial for normal keratinocytes function which support wound healing and tissue regeneration [28,29]; as, during the administration, an increased concentration of BP in these tissues is likely.

## 5. Bisphosphonates Influence Oral Wound Healing

Besides the direct cytotoxic effect on fibroblasts and keratinocytes, different research groups have detected a direct inhibition of wound healing and impaired mucosa function. The development of gastric erosion and ulcers is a well described side effect of BPs. Several studies demonstrated that especially nitrogen-containing BPs show a negative effect on different gastric cell types. Wallace et al. showed, in an ex vivo gastric chamber model, that the gastric mucosa is inhibited by nitrogen-containing BPs [30]. Landesberg et al. showed that bisphosphonate pre-treatment of oral mucosal cells inhibits proliferation and wound healing at clinically relevant doses and that this inhibition is not due to cellular apoptosis [31].

## 6. Summary

The development and maintenance of BRONJ is a multifactorial event. The adverse impact of BPs results in inhibition of cellular function of the hard tissue, as well as inhibitory effects of the mucosal layer. Inhibition of fibroblasts and keratinocytes leads to disturbed integrity of the mucosal layer and has a negative influence on bone metabolism via the RANK-L-OPG-system. Besides this, the mucosal immune system is compromised and vulnerable to infection. Further, the mucosal architecture is influenced by BPs and results in mucosal thinning.
